# Supplementary material for: Reversible Thiol Oxidation Inhibits the Mitochondrial ATP Synthase in Xenopus laevis Oocytes
Source: Antioxidants (Basel). 2020 Mar 5;9(3):215. doi: 10.3390/antiox9030215 (PMC7139892; doi:10.3390/antiox9030215)
Supplement: Supplementary file 1 [file antioxidants-09-00215-s001.pdf]

Supplementary Table 1. Manufacturer and catalogue number by material reagent used.

CHEMICAL/REAGENT	MANUFACTURER	CATALOGUE NUMBER
Protease inhibitor tablet	Sigma, UK.	11697498001
Fatty acid free BSA	Sigma, UK.	A8806
Alamethicin	Sigma, UK.	A4665
ATP	Acros Organics, UK.	102800100
PEP	Alfa Aesar, UK.	B20358
PK	Sigma, UK.	P1506
LDH	Sigma, UK.	L2500
NADH	Sigma, UK.	10107735001
Antimycin A	Sigma, UK.	A8674
$Pb(NO_3)_2$	Sigma, UK.	203580
Diphenyleneiodonium	Enzo Life Sciences, UK,	BML-CN240-0010
Imidazole	VWR, UK.	0527
Tricine	Acros Organics, UK.	172642500
Tris	Fisher Chemicals, UK.	BP152-1
Glycine	Fisher chemicals, UK.	G/800/60
$MgSO_4$	Sigma, UK.	M2643
Oligomycin	Sigma, UK.	04876
DOC	Sigma, UK.	30970
DDM	Sigma, UK.	D5172
NEM	Sigma, UK.	E3876
TCO-PEG4-NHS	Jena Bioscience, Germany.	CLK-A137-10
Tetrazine Agarose	Jena Bioscience, Germany.	CLK-1199-2
Biotin-dPEG®3-MAL	Sigma, UK.	QBD10201
Streptavidin, Alexa Fluor™ 647 conjugate	Thermofisher Scientific, UK.	S21374
6000 kDa spin column	BioRad, UK.	7326222
TCEP	Thermofisher Scientific, UK.	T2556
DTT	VWR, UK.	443853B
TCO-PEG3-NEM	Click Chemistry Tools, USA.	1002
Tz-PEG5	Click Chemistry Tools, USA.	1090
Precast 4-15% gels.	BioRad, UK.	4561085
PVDF membrane	BioRad, UK.	1620261
Anti-ATP-a-F_1 primary	Abcam, UK.	ab14748
Alexa Fluor$^{(R)}$ 750 mouse secondary antibody	Abcam, UK.	ab175741
